# Illegal Abortion in Iran: Challenges, Consequences, Recommendations

**DOI:** 10.34172/aim.34080

**Published:** 2025-06-01

**Authors:** Habibollah Azarbakhsh, Mahdiyeh Rashedi

**Affiliations:** ^1^Department of Biostatistics and Epidemiology, School of Health, Ahvaz Jundishapur University of Medical Sciences, Ahvaz, Iran; ^2^Deputy of Health, Ahvaz Jundishapur University of Medical Sciences, Ahvaz, Iran

## Dear Editor,

 Abortion is defined as terminating pregnancy before the fetus becomes viable. Global statistics show that between 2015 and 2019, 121 million unintended pregnancies occurred annually worldwide, and 61% of them resulted in an abortion.^1^ Prohibition of abortion has been codified from ancient time in documents such as the “Hippocratic Oath” and is claimed as “an almost absolute value” in human history.^2^ Unsafe abortions are one of the leading causes of mortality and morbidity among women in developing countries.^3^ In Iran, abortion was first permitted in 1997 for cases of thalassemia major and fetal encephalopathy before the 19th week of pregnancy.^4^ Due to the sensitive nature of this phenomenon, there are no accurate statistics on the number of abortions in Iran. It is estimated that 300 000 to 600 000 illegal abortions occur in Iran annually.^5^ The abortion rate in Iran is estimated at one in four women.^6^ With the approval of the Law on Family and Youth Protection in Iran in 2021 aiming to increase the population, strict security laws were passed against abortion providers.^7^ In a study conducted by Haseli et al in 2016 in Iran, financial hardship and uncertainty about the future, pursuit of a prosperous life, unstable marital relationship, health and fertility issues and cultural factors were reported as the main causes of abortio.^7^ The results of a study showed that women with a diploma or higher education were less likely to report having had an abortion.^8^

 Despite the fact that unsafe abortion plays a significant role in mortality and morbidity among women of reproductive age, there is little research on the consequences of abortion.^9^ In one of the few studies conducted on the consequences of abortion in Iran, anxiety, depression, and feelings of guilt were among its consequences.^10^

 The following are recommended to prevent illegal abortions in Iran: Informing people about the dangerous physical, mental, and psychological harms caused by abortion, promoting having more children as a value, reducing the economic and social problems of mothers, requiring Ministry of Health employees, especially doctors and midwives, to re-train on the legal and ethical issues of abortion from an Islamic perspective, regular and intrusive inspections of pharmacies, private clinics, and any health care provider that may perform or provide illegal abortions, regularizing tests and examinations during pregnancy, preventing extensive screening without indication, and minimizing the error rate of these tests, providing conditions for young people to marry to reduce illegitimate relationships, and promoting abstinence from alcohol or illegal drugs during pregnancy.

## References

[1] Rashidpouraie R, Vahid Dastjerdi M, Shojaei A, Saeeditehrani S, Sharifi M, Joodaki K (2021). Complications of illegal abortion in the suburbs of Tehran: a 9-year cross-sectional study. J Res Med Sci.

[2] Abbasi M, Shamsi Gooshki E, Allahbedashti N (2014). Abortion in Iranian legal system: a review. Iran J Allergy Asthma Immunol.

[3] Bearak J, Popinchalk A, Ganatra B, Moller AB, Tunçalp Ö, Beavin C (2020). Unintended pregnancy and abortion by income, region, and the legal status of abortion: estimates from a comprehensive model for 1990-2019. Lancet Glob Health.

[4] Mahdavi SA, Jafari A, Azimi K, Dehghanizadeh N, Barzegar A (2020). Therapeutic abortion in Iran: an epidemiologic study of legal abortion in 2 years. BMC Res Notes.

[5] Shirdel E, Asadisarvestani K, Hami Kargar F (2024). The abortion trend after the pronatalist turn of population policies in Iran: a systematic review from 2005 to 2022. BMC Public Health.

[6] Fallahian M, Tavana S (2022). Trends in techniques of abortion in Iran from 1994 to 2014. J Obstet Gynecol Cancer Res.

[7] Haseli A, Rahnejat N, Rasoal D (2024). Reasons for unsafe abortion in Iran after pronatalist policy changes: a qualitative study. Reprod Health.

[8] Hosseini H, Erfani A, Nojomi M (2017). Factors associated with incidence of induced abortion in Hamedan, Iran. Arch Iran Med.

[9] Singh S (2010). Global consequences of unsafe abortion. Womens Health (Lond).

[10] Hosseini-Chavoshi M, Abbasi-Shavazi MJ, Glazebrook D, McDonald P (2012). Social and psychological consequences of abortion in Iran. Int J Gynaecol Obstet.

